# *Pseudoproleptus* sp. Larvae (Nematoda: Cystidicolidae) parasitizing *Macrobrachium amazonicum* (Decapoda: Crustacea) in the state of Pará, Northern Brazil

**DOI:** 10.1590/S1984-29612025042

**Published:** 2025-08-07

**Authors:** Patrick José Colares Cardoso, Liane Rodrigues Galvão de Cristo, Yan Rafael Gillet Santa Brigida, Elane Guerreiro Giese, Raul Henrique da Silva Pinheiro

**Affiliations:** 1 Laboratório de Histologia e Embriologia Animal, Instituto da Saúde e Produção Animal, Universidade Federal Rural da Amazônia – UFRA, Belém, PA, Brasil; 2 Programa de Pós-graduação em Saúde e Produção Animal na Amazônia, Instituto da Saúde e Produção Animal, Universidade Federal Rural da Amazônia – UFRA, Belém, PA, Brasil

**Keywords:** Helminth, nematode, parasite, shrimp, Guamá river, Helminto, nematoide, parasito, camarão, Rio Guamá

## Abstract

Third-stage larvae of a nematode species were found encapsulated in the cephalothorax of *Macrobrachium amazonicum* from the Guamá River, municipality of Belém, in the state of Pará, northern Brazil. Of a total of 120 specimens of *M. amazonicum* that were caught, 15.8% were parasitized with Cystidicolidae larvae that had morphological characteristics compatible with the genus *Pseudoproleptus*, such as rounded cephalic end, two pseudolips, slit-shaped buccal opening with four large submedian lips, vestibule with prostom and conical tail with a small mucron. The low presence of *Pseudoproleptus* larvae did not affect the growth of the shrimp, but these results cannot be generalized since the sampling was relatively low. Therefore, so the occurrence of this nematode could still negatively affect the growth and reproduction of these shrimp, leading to their death, which could directly affect their population dynamics and consumption by the Amazonian riverside population. Finally, we recommend more analyses with a greater sampling effort.

## Resumo

Larvas de terceiro estágio de uma espécie de nematódeo foram encontradas encapsuladas no cefalotórax de *Macrobrachium amazonicum* do Rio Guamá, município de Belém, no estado do Pará, norte do Brasil. Em um total de 120 espécimes de *M. amazonicum* capturados, 15,8% estavam parasitados por larvas de Cystidicolidae que apresentavam características morfológicas compatíveis com o gênero *Pseudoproleptus*, como extremidade cefálica arredondada, dois pseudolábios, abertura bucal em forma de fenda com quatro grandes lábios submedianos, vestíbulo com prostom e cauda cônica com um pequeno mucro. A baixa presença de larvas de *Pseudoproleptus* não afetou o crescimento dos camarões, porém estes resultados não podem ser generalizados uma vez que a amostragem foi relativamente baixa. Portanto, a ocorrência desse nematódeo poderia afetar negativamente o crescimento e a reprodução dessa espécie de camarão, levando-o à morte, o que poderia afetar diretamente sua dinâmica populacional e o consumo pela população ribeirinha da Amazônia. Desta forma, recomendamos mais análises com um esforço amostral maior.

## Introduction

The Amazon biome, encompassing a vast geographical area and a diverse mosaic of habitats including one of the planet’s most biodiverse environments (Pimentel, 2003). The freshwater shrimp family Palaemonidae stands out as one of the most diverse taxa, with approximately 981 species described. Notably the genus *Macrobrachium* Spence Bate 1868 exhibits significant versatility among the biological communities in aquatic ecosystems. These shrimps play a major in ecological processes, functioning at various levels of the trophic chain (Rodd & Reznick, 1991; Lima et al., 2014).

The genus *Macrobrachium* comprises 233 species worldwide, of which only 45 are found on the American continent (De Grave & Fransen, 2011) and seven species have been recorded in the state of Pará, Brazil (Pimentel & Magalhães, 2014; Quaresma & Martinelli-Lemos, 2020). *Macrobrachium amazonicum* (Heller, 1862) popularly known as “Camarão do Rio Amazonas - Amazon River shrimp” or “Camarão Canela - Cinnamon Shrimp” is originally endemic to the Amazonian rivers of South America that flow into the Atlantic Ocean (Holthuis, 1952; Maciel & Valenti, 2009). This species is considered one of the most economically important freshwater crustaceans in Brazil due to its exploitation through small-scale fishing, particularly in the northern region. This activity provides a vital source of protein and income for both riverside and urban populations (Lucena-Frédou et al., 2010; Bentes et al., 2011).

While crustacean fishing holds significant social and commercial importance for the northern region of Brazil, limited data exists regarding parasitism in Amazonian shrimp. The few studies available conducted by Moravec & Santos (2009) and Melo et al. (2011), reported infections by larvae of Cystidicolidae nematodes in *M. amazonicum*.

This knowledge of the parasitic fauna of *M. amazonicum* is important as a preventive measure for management and quality of this shrimp species, which is intensively used by the regional population as a high protein food source. Thus, the aim of this study was to describe the occurrence, ecological attributes of parasitism and morphology of larvae of Cystidicolidae nematodes parasitizing *M. amazonicum*, and to evaluate whether these larvae affect the shrimp's body condition and growth in the state of Pará, northern Brazil.

## Material and Methods

### Fish and collection location

One hundred and twenty *M. amazonicum* (50 females and 70 males) specimens were obtained by fishers in the Guamá River, in the municipality of Belém (01º 27' 21” S, 48º 30' 16” W), state of Pará, Brazil. The shrimps were transported in an isothermal box to the Laboratório de Histologia e Embriologia Animal, Instituto de Saúde e Produção Animal, Universidade Federal Rural da Amazônia, City of Belém for necropsy. After biometric parameter analyses for total length, cephalothorax length and abdominal length (see Table 1), the shrimps were necropsied for analyses of parasite helminths.

**Table 1:** Descriptive statistics of biometric parameters of *Macrobrachium amazonicum* collected in Guamá River, Pará state, northern Brazil.

**Sex**	**Biometric parameter**	**Min.**	**Max.**	**x̄**	**±SD**	**Median**
**♀+♂**	TL (cm)	3.39	8.91	6.34	1.13	6.26
	CL (cm)	1.38	4.49	2.80	0.58	2.73
	AL (cm)	2.01	4.9	3.55	0.63	3.56
	Weight (g)	0.64	5.7	2.41	1.05	2.27
**♂**	TL (cm)	3.39	8.63	6.35	1.17	6.31
	CL (cm)	1.38	3.94	2.80	0.55	2.73
	AL (cm)	2.01	4.9	3.55	0.67	3.64
	Weight (g)	0.64	5.7	2.41	1.06	2.29
**♀**	TL (cm)	4.35	8.91	6.34	1.08	6.22
	CL (cm)	1.71	4.49	2.79	0.61	2.75
	AL (cm)	2.3	4.81	3.55	0.58	3.55
	Weight (g)	0.8	5.02	2.42	1.04	2.07

TL: Total length; CL: Cephalothorax length; AL: Abdominal length. ♀: Female; ♂: Male. Min.: minimum values; Max.: maximum values. x̅ = mean; ±SD = standard deviation.

### Collection, fixation and identification procedures of parasites

The cephalothorax and abdomen of each specimen was isolated in a Petri dish containing a sodium chloride solution (0.9%) and analyzed using a stereomicroscope (LEICA-ES2). The nematode larvae found dead were fixed in AFA solution (93 parts 70% ethyl alcohol, 5 parts formaldehyde, and 2 parts glacial acetic acid) and stored at room temperature. For morphological and morphometric analysis ten larvae were dehydrated in an ethanol series, clarified with lactophenol, placed on a microscope slide under a coverslip as a temporary mount, observed using a light microscope, and photographed using a microscope (LEICA DM2500) with an imaging capture system. Measurements are given in micrometers unless otherwise noted and are presented as the mean followed by the range (minimum and maximum values) in parentheses.

For scanning electron microscopy, six larvae were washed in phosphate-buffered saline (pH 7.0), post-fixed in 1% osmium tetroxide, dehydrated to the critical point of CO_2_, metalized with gold-palladium, and analyzed with scanning electron microscope (VEGA 3 LMU/TESCAN) at the Laboratório de Microscópia Eletrônica de Varredura, Instituto da Saúde e Produção Animal - Universidade Federal Rural da Amazônia - UFRA, state of Pará, Brazil.

The ecological terms of parasitism used (*i.e.*, prevalence, mean intensity and mean abundance) were according to Bush et al. (1997) and Bautista-Hernández et al. (2015). Taxonomic classification of *M. amazonicum* was in accordance with Cervigón et al. (1992), and sexual differentiation was determined by morphological characteristics according to Valenti (1996) and taxonomic classification of nematodes was in accordance with Moravec & Santos (2009), Melo et al. (2011).

Specimen deposit: Four specimens (MPEG-NEM 000418) were deposited in the Coleção de Invertebrados of the Museu Paraense Emílio Goeldi (MPEG), Belém, Pará, Brazil.

### Statistical analysis

The relationship between total length and parasitic intensity (number of helminths) was analyzed using a generalized linear model (GLM) with a Negative Binomial distribution, implemented via the MASS package (Ripley et al., 2014) in R. This approach was chosen due to the presence of overdispersion in the count data, as identified in the Poisson model (dispersion > 1). Model fit quality was assessed using McFadden’s pseudo-R^2^ (McFadden, 1973). The size frequency (Total length, TL, in mm) was grouped into 0.5 cm classes and plotted as histograms. To assess the body condition of parasitized and non-parasitized shrimp, we used the residuals from a log-log linear regression model between body weight and total length (TL), an approach suitable for cases of allometric growth. As noted by Sánchez et al. (2018), condition indices are useful for analyzing infection-fitness relationships.

A t-test was used to compare the size and body condition of parasitized and non-parasitized shrimp, considering males and females separately, while the Mann-Whitney test was used to assess differences in the abundance and intensity, as this dataset did not meet the assumptions of normality and homoscedasticity. To determine the influence of parasitism on shrimp growth, a linear regression was performed on the log-transformed weight (g) against the log-transformed total length. An analysis of covariance (ANCOVA) was then used to evaluate differences in growth between parasitized and non-parasitized shrimp.

Before conducting the statistical analysis, all data were assessed for normality using the Shapiro-Wilk test and for homoscedasticity using Levene's test. All hypotheses were evaluated with an adopted significance level of α = 0.05 (Zar, 2010). The analyses were performed using R software version 4.4.1 (R Core Team, 2024).

## Results

### Morphological and morphometric data

A total of 74 larvae of nematodes were recovered from *M. amazonicum*. All specimens collected showed characteristics compatible with third-stage larvae of *Pseudoproleptus* sp. (Nematoda: Cystidicolidae). The parasites were found encapsulated in the cephalothorax of *M. amazonicum*. The morphological and morphometric characteristics of the larvae of *Pseudoproleptus* sp. are presented below.

Nematoda (Rudolphi, 1808)

Family Cystidicolidae Skrjabin, 1946

*Pseudoproleptus* Khera, 1955

**Figure 1:**
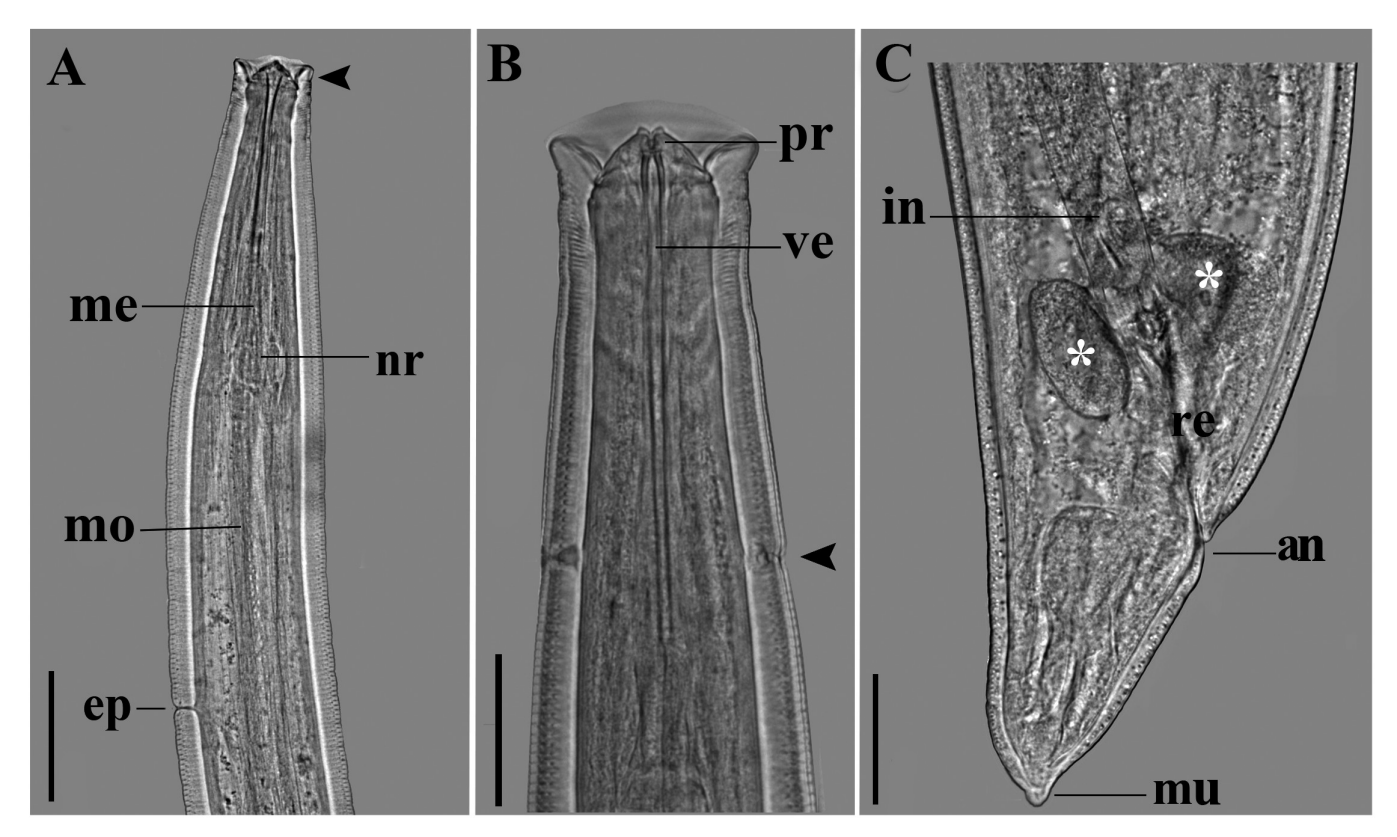
Third-stage larva of *Pseudoproleptus* sp. from *Macrobrachium amazonicum*, commercialized in Pará state, northern coast of Brazil: **(A)** Anterior lateral view, cephalic helmet-like cuticular structure (arrowhead), nerve ring (nr), muscular esophagus (mo) and excretory pore (ep). Bar = 100 μm; **(B)** Detail of prostom (pr), vestibule (ve) and deirids (arrowheads). Bar = 50 μm; **(C)** Posterior portion, portion of the intestine (in), rectum (r), unicellular rectal glands (*), anus (an), the tail with mucron (mu). Bar = 50 μm.

**Figure 2:**
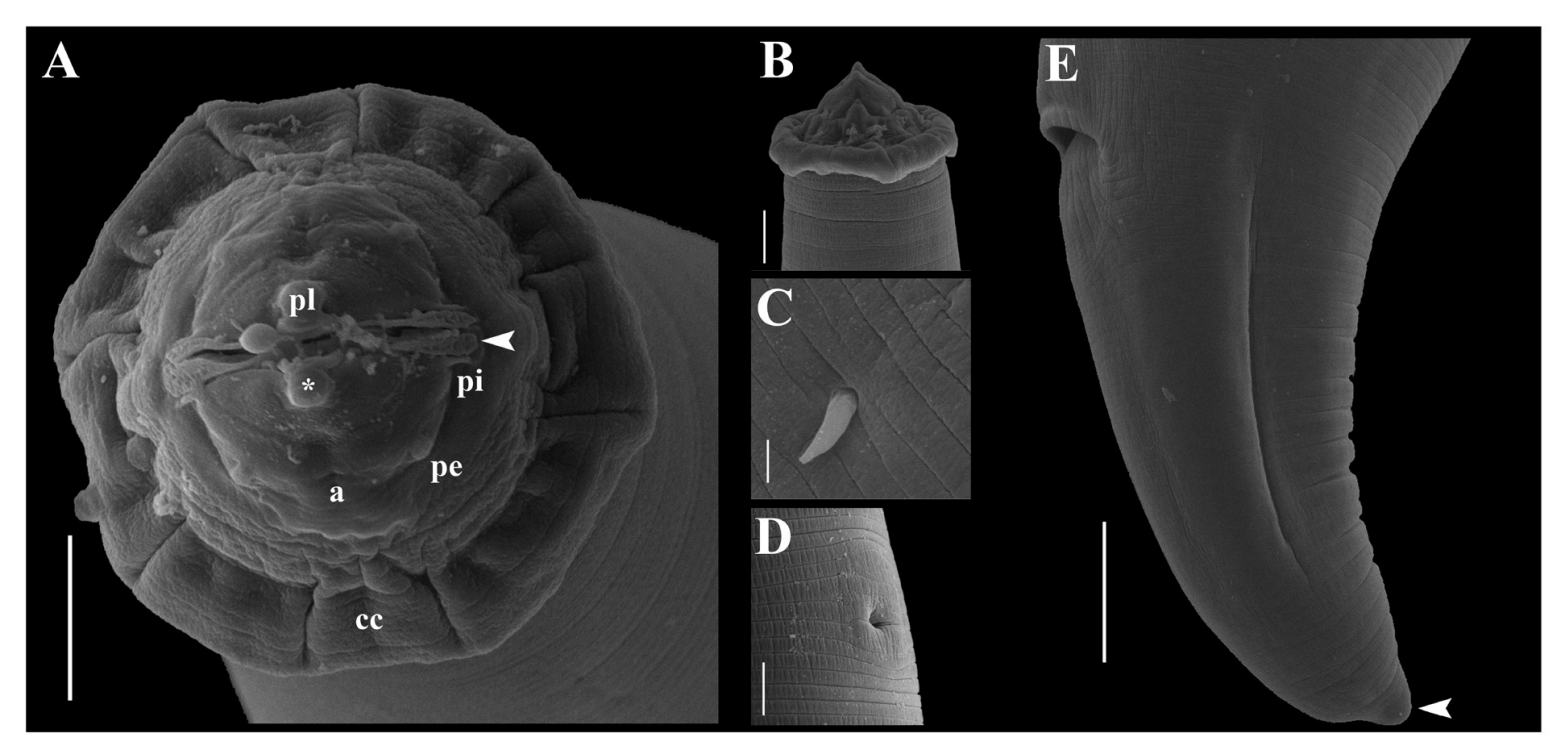
Third-stage larva of *Pseudoproleptus* sp. from *Macrobrachium amazonicum*, SEM micrographs: **(A)** Cephalic apical view: amphid (a), cephalic papilla of internal circle (pi); cephalic papilla of external circle (pe), cephalic helmet-like cuticular structure (cc), pseudolabium (pl), pseudolabial terminal protrusion (*), outer, elongate part of sublabium (arrowhead). Bar = 10 μm; **(B)** Anterior end of body, lateral view cephalic helmet-like cuticular structure. Bar = 10 μm; **(C)** Deirid views. Bar = 2 μm; **(D)** Excretory pore. Bar = 10 μm; **(E)** Tail, lateral view, with small knob-like terminal projection (arrowhead). Bar = 20 μm.

*Pseudoproleptus* sp. (Description based on ten third-stage larvae) (Figures 1 and 2)

Medium-sized nematodes, body filiform, measuring 22 mm (17‒28 mm), maximum width at esophageal/intestinal junction 184 (143‒229). Cuticle with thick transverse striations (Figures 1A, 2B, C). Anterior end of body with cephalic helmet-like cuticular structure 25 (20‒32) long; 69 (45‒93) wide (Figures 1A, 2A). Slit-like oral aperture with four large submedian labia (2 dorsolateral and 2 ventrolateral) (Figure 2A). Sublabia plate-like, narrowed proximally, widening to distal ends. Lateral pseudolabia small, with terminal protrusion (Figure 2B). Eight submedian cephalic papillae arranged in two circles and a pair of lateral amphids present: four papillae of external circle larger than those of internal circle. Vestibule with prostom 157 (120‒193) long (Figure 2B). Length of muscular esophagus 0.95 mm (0.83‒1.2 mm) long; 25 (12‒35) wide; length of glandular esophagus 4 mm (3‒4 mm) long; 67 (47‒83) wide. Length ratio of muscular and glandular parts of esophagus 1:5 (1:4‒6). Length of entire esophagus and vestibule with prostom represents 26% (19−38%) of whole-body length. Deirids, nerve-ring and excretory pore at 137 (110‒167) (Figures 1B, 2C), 211 (165‒257) (Figure 1A) and 448 (340‒557) (Figures 1A, 2D), respectively, from anterior extremity. Genital primordium indistinct. Rectum is a short hyaline tube; three small, unicellular rectal glands are present (Figure 1C). Tail conical, 118 (102–163) long, with small knob-like terminal projection 7 (5–8) long (Figures 1C, 2E).

### Parasitological data

**Figure 3:**
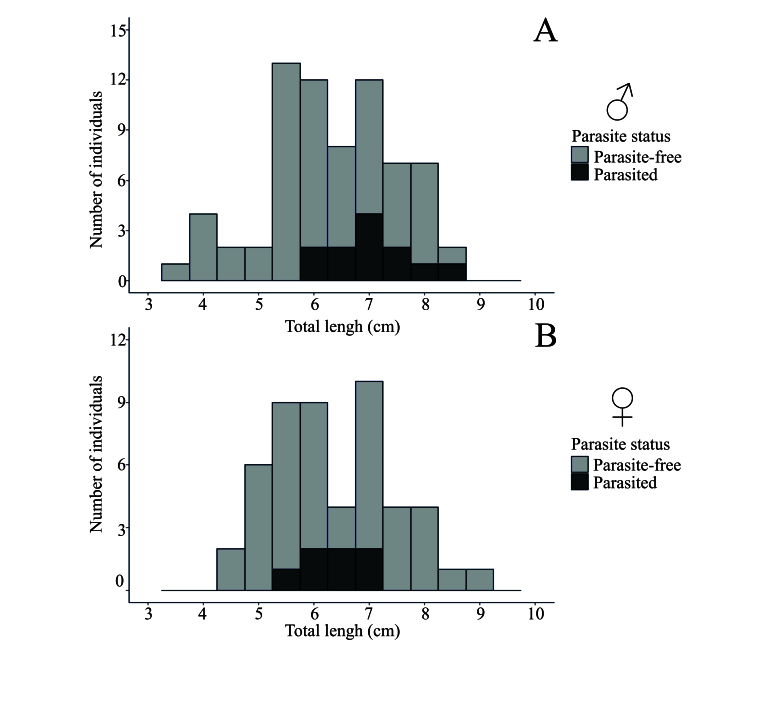
Histograms showing the total length classes of male (**A**) and female (**B**) *Macrobrachium amazonicum*.

No significant differences were found in the residuals from the log-log linear regression model, cephalothorax size, or weight, between parasitized and non-parasitized shrimp, considering both males and females. Additionally, there was no significant difference in total length between parasitized and non-parasitized females (Table 2). However, a significant difference in total length was observed when both sexes were analyzed together (t = -2.53, p = 0.02) and when only males were considered (t = -3.10, p < 0.01) (see Table 2). The ecological attributes of parasitism can be seen in Table 3. The result of the negative binomial model of the relationship between the intensity of larvae recovered from *M. amazonicum* and the total length of the hosts, indicated no significant association (Pseudo-R^2^ = 0.4938; β = 0.0046; 95% CI: [−0.578, 0.595]; p = 0.987). Therefore, parasitism was present in individuals from 5.34 cm (Figure 3A). One female ovigerum had a longer length of 6.76 cm (Figure 3B) and higher intensity of infection with 21 third-stage larvae of *Pseudoproleptus* sp. No significant difference between intensity and abundance of parasites was found between males and females. The analysis of covariance (ANCOVA) showed no significant difference in relative growth between non-parasitized and parasitized shrimp (F = 0.0202, p = 0.88).

**Table 2:** Parasitological indices of larvae of *Pseudoproleptus* sp. harvested from *Macrobrachium amazonicum* from Guamá River, in Pará state northern Brazil.

**Sex**	**P (%)**	**MI**	**±SD**	**MA**	**±SD**	**R**
**♀+♂**	15.8	3.9	4.85	0.6	2.36	1–21
**♀**	14.0	5.8	7.47	0.8	3.32	1–21
**♂**	18.0	2.7	2.05	0.5	1.32	1–7

P: Prevalence; MI: Mean intensity; MA: Mean abundance; ±SD: standard deviation; R: Range. ♀: Female; ♂: Male.

**Table 3:** Results of the t-test and Mann-Whitney of biometric parameters between, males, females, parasitized and parasite-free shrimps.

	**TL (cm)**			**CL (cm)**			**Weight (g)**		
	**t**	**p**	**CI**	**t**	**p**	**CI**	**w**	**p**	**CI**
**♂_f_** × **♂_i_**	-3.1	**0.01**	[-1.4584, -0.2896]	-2.01	0.06	[-0.70525, 0.0189]	270	0.23	[-1.2390, 0.1999]
**♀_f_** × **♀_i_**	-0.09	0.93	[-0.6442, 0.5935]	-0.07	0.94	[-0.33076, 0.3083]	152	0.98	[-0.7800, 0.9200]
**♂** × **♀**	-0.03	0.98	[-0.4152, 0.4044]	-0.07	0.94	[-0.2238, 0.2077]	1771	0.91	[-0.3900, 0.3599]
**♀♂_f_** × **♀♂_i_**	-2.53	**0.02**	[-0.9971, -0.1094]	-1.76	0.08	[-0.47142, 0.0353]	823	0.33	[-0.8000, 0.2401]

TL: Total length; CL: Cephalothorax length. ♀: Female; ♂: Male. f: parasite-free; i: infected with parasite.

## Discussion

The nematodes found encapsulated in the cephalothorax of *M. amazonicum* captured in the Guamá River, presented morphological characteristics compatible with nematodes of the family Cystidicolidae (Skrjabin, 1946) of genus *Pseudoproleptus* Khera, 1953, including the presence of a helmet-like cuticular structure in the anterior extremity, slit-like oral aperture with 4 large submedian labia, vestibule with prostom, muscular and glandular esophagus and conical tail with small knob-like terminal projection. Morphologically and morphometrically the third-stage larvae *Pseudoproleptus* sp. parasite of *M. amazonicum*, *Ageneiosus ucayalensis* Castelnau, 1855 (Siluriformes: Auchenipteridae) and *Satanoperca jurupari* (Heckel, 1840) (Cichliformes: Cichlidae) present similarities between each other.

One morphologically important detail is that the specimens of *Pseudoproleptus* sp. observed in the present study bears tapered deirids, which differs from that observed by Melo et al. (2011) who described bifurcated deirids in *Pseudoproleptus* sp. recovered from *S. jurupari* in the same locality as this study. Melo et al. (2011) claims that the *Pseudoproleptus* sp. collected from *S. jurupari* shows forked deirids, but his SEM observation of the deirids of one specimen shows a broken end with only evidence of a double root in this structure.

For Moravec et al. (2003), identifying Cystidicolidae larvae is generally problematic, because the development and larval morphogenesis of the species described remain unknown. Moravec (2007) further states that the adult taxonomy for the Spirurine families is unsatisfactory. Morphological and morphometric comparison of third-stage larvae of *Pseudoproleptus* sp. parasites of *M. amazonicum* in the state of Pará, are compared to the work of Moravec & Santos (2009) and Melo et al. (2011) and are presented in the Table 4.

**Table 4:** Morphological and morphometric comparison of third-stage larvae of *Pseudoproleptus* sp. in *Macrobrachium amazonicum* from Guamá River, in the state of Pará, Brazil.

**Caracteres**	**Third-stage larvae of *Pseudoproleptus* spp.**			
**Group of host**	**Decapoda**		**Cichliformes**	**Siluriformes**
**Family**	**Palaemonidae**		**Cichlidae**	**Auchenipteridae**
**Hosts**	***Macrobrachium amazonicum***		***Satanoperca jurupari***	***Ageneiosus*** ***ucayalensis***
**Locality**	**Guamá River,** **state of Pará**	**Mexiana Island,** **state of Pará**	**Guamá River,** **state of Pará**	
**Prevalence**	-	32%	0.90%	0.90%
**Length** ^mm^	17‒28	19.71‒25.65	16.20‒21.63	20
**Width**	143‒229	163‒190	140‒180	96
**Cephalic helmet-like** ^L^	20‒32	‒	30‒50	‒
**Cephalic helmet-like** ^W^	45‒93	‒	45‒85	‒
**Vestibule with prostom ^L^**	120‒193	165‒189	112‒175	125
**Deirids**	110‒167	174‒195	110‒200	‒
**Nerve ring**	165‒257	249‒282	180‒285	‒
**Excretory pore**	340‒557	530‒558	412‒482	‒
**Muscular esophagus ^L, mm^**	0.829‒1.2	0.804‒0.930	0.640‒1	1.053
**Muscular esophagus ^W^**	12‒35	15‒21	20‒50	‒
**Glandular esophagus ^L, mm^**	3‒4	3.66‒4.27	3.20‒3.90	4.03
**Glandular esophagus ^W^**	47‒83	57‒66	50‒70	‒
**Tail**	102‒163	135‒180	100‒145	‒
**Mucron**	5‒8	3‒6	‒	‒
**References**	From this study	Moravec & Santos (2009)	Melo et al. (2011)	

^mm^: millimeters; ^L^: length; ^W^: width.

The lack of significant differences in body condition, cephalothorax size, and weight between parasitized and non-parasitized *M. amazonicum* shrimp suggests that infection by *Pseudoproleptus* sp. larvae does not directly impact the physical condition or growth of the shrimp. This may indicate that, at the observed intensity and prevalence, helminth parasites are not sufficiently pathogenic to affect shrimp growth or overall condition.

Parasitism was observed only in males larger than 5.89 cm and females between 5.34 cm and 7.2 cm, aligning with reproductive maturity for the species, which is reached at 4.5-6 cm (Maciel & Valenti, 2009; Pantaleão et al., 2011). This restriction suggests that *Pseudoproleptus* sp. larvae target only reproductively mature shrimps. The absence of significant growth differences between parasitized and parasite-free females (Table 3) supports this conclusion, indicating that parasitism does not severely impact female growth but rather occurs within a specific size range, likely due to ecological or physiological factors. The focus on reproductively sized individuals may reflect the greater exposure, greater energy availability, and changes in immune function during reproductive phases.

Spatial-temporal variation in *M. amazonicum* populations, as reported by Bentes et al. (2011), showed a higher abundance of males near creek mouths in March (rainy season) and a concentration of females in headwater sites in September (dry season). This variation could influence differing exposure to parasites for males and females. Ibrahim et al. (2021) also observed that larger, dominant morphotype “Green Claw” males exhibit aggressive behavior toward smaller morphotypes, potentially increasing their exposure to parasites. Another hypothesis is that the aggressive nature of larger males reduces the survival rates of smaller shrimp, making larger hosts more susceptible to helminth parasites due to their higher survival and extended lifespan.

In Brazil there are few records of parasitism in crustaceans. In this study, 15.8% of *M. amazonicum* were parasitized by *Pseudoproleptus* sp. larvae. In the same biogeographic region of this study, different authors also reported parasitism by *Pseudoproleptus* sp. in shrimps and fishes. Moravec & Santos (2009) described the occurrence and prevalence of 32% by *Pseudoproleptus* sp. larvae in *M. amazonicum* on Mexiana Island, in state of Pará (Brazil). Melo et al. (2011) demonstrated a low prevalence (0.9%) of parasitism by *Pseudoproleptus* sp. in *A. ucayalensis*. Melo et al. (2011) reported the highest prevalence of 37% of parasitism by *Pseudoproleptus* sp. larvae in *S. jurupari* caught in the Guamá River, state of Pará. Previously, Takemoto & Lizama (2010) noted the low specificity of nematodes for hosts, especially in the larval stage. Therefore, there seems to be a wide distribution of *Pseudoproleptus* sp. larvae in the state of Pará.

Parasitism by *Pseudoproleptus* sp. larvae in commercially important shrimp and fishes in the state of Pará demonstrates that the diversity of parasites in this region is still poorly known. According to Moravec & Santos (2009), no adult species of *Pseudoproleptus* has been described in the Americas so far. These same authors, when describing the morphology of *Pseudoproleptus* sp. larvae recovered from *M. amazonicum* in the state of Pará, related these larvae to *Pseudoproleptus izecksohni* (Fabio, 1982). However, *P. izecksohni* was inappropriately proposed to the *Heliconema* genus as *Heliconema izecksohni* Fabio, 1982, a parasite of *Hoplias malabaricus* (Bloch, 1794) (Characiforme: Erythrinidae) caught in the municipality of Campos, Rio de Janeiro state, based on morphological and freshwater host data (see Moravec et al., 2003). In addition, the same authors highlight that *Heliconema* species are parasites of marine fish, especially of the anguilliform order. Therefore, Moravec et al. (2008) transfers *H. izecksohni* to the genus *Cystidicoloides*. Luque et al. (2011) when listing the nematodes associated with fishes in Brazil accepted this species as *Cystidicoloides izecksohni* (Fábio, 1982).

The occurrence of *Pseudoproleptus* sp. larvae in different hosts in the Guamá river demonstrates the presence of a complex life cycle of this parasite in the Pará estuary. There are few data on the life cycle of nematodes of the family Cystidicolidae (Anderson, 2000; Moravec, 2007; Melo et al., 2011). Freshwater crustaceans such as *M. amazonicum* and aquatic insects, function as primary intermediate hosts, with fish being the secondary intermediate hosts (Moravec, 2007). Melo et al. (2011) reported that some species of fish may participate in the life cycle as paratenic hosts, as has been reported for *A. ucayalensis* and *S. jurupari*, which were parasitized by third-stage larvae of *Pseudoproleptus* sp.

Pinheiro et al. (2019), records the presence of *Pseudoproleptus* sp. larvae in *Astronotus ocellatus* (Agassiz, 1831) (Cichliformes: Cichlidae) captured in the Tapajós River (Brazil) and used as food in the municipality of Santarém, the same biogeographical region of this study. Therefore, this cichlid is also an important intermediate host for *Pseudoproleptus* sp. larvae in eastern Amazon.

## Conclusions

The occurrence of larvae of *Pseudoproleptus* in *M. amazonicum* indicates that this crustacean acts as an intermediate host for third-stage larvae of this genus in the Guamá River. Although the prevalence of parasitism was low, and the differences in growth between parasitized and non-parasitized individuals were not significant, these results cannot be generalized since the sampling was relatively low, therefore the occurrence of this nematode in *M. amazonicum* can still negatively affect the growth and reproduction of this shrimp, leading it to death, directly affecting its population dynamics, for this we recommend more analysis with a larger sample *n* .

The occurrence of larvae reinforces the presence of a complex life cycle for *Pseudoproleptus* in the estuarine region of Pará, with the definitive host still unknown. In addition, because this Amazon shrimp is important to the region's population as a source of foreign exchange, due to its trade and high potential for cultivation, sanitation in aquaculture systems is a critical factor in its production.

## Data Availability

Data will be made available on request.
